# Link between gut microbiota dysbiosis and childhood asthma: Insights from a systematic review

**DOI:** 10.1016/j.jacig.2024.100289

**Published:** 2024-06-12

**Authors:** Rabbiya Aslam, Laura Herrles, Raquel Aoun, Anna Pioskowik, Agata Pietrzyk

**Affiliations:** Scientific Group of Microbiology and Parasitology and the Department of Microbiology, Faculty of Medicine, Jagiellonian University Medical College, Kraków, Poland

**Keywords:** Asthma, gastrointestinal microbiome, infant, newborn, gut microbiota

## Abstract

Asthma, a chronic inflammatory disorder of the airways, is a prevalent childhood chronic disease with a substantial global health burden. The complex etiology and pathogenesis of asthma involve genetic and environmental factors, posing challenges in diagnosis, severity prediction, and therapeutic strategies. Recent studies have highlighted the significant role of the gut microbiota and its interaction with the immune system in the development of asthma. Dysbiosis, an imbalance in microbial composition, has been associated with respiratory diseases through the gut–lung axis. This axis is an interaction between the gut and lungs, allowing microbial metabolites to influence the host immune system. This systematic review examines the association between gut microbiota composition, measured using 16S rRNA sequencing, during infancy and childhood, and the subsequent development of atopic wheeze and asthma. The results suggest that higher alpha diversity of bacteria such as *Bifidobacterium, Faecalibacterium*, and *Roseburia* may have protective effects against asthmatic outcomes. Conversely, lower relative abundances of bacteria like *Bacteroides* and certain fungi, including *Malassezia*, were associated with asthma. These findings highlight the potential of early screening and risk assessment of gut microbiota to identify individuals at risk of asthma. Furthermore, investigations targeting gut microbiota, such as dietary modifications and probiotic supplementation, may hold promise for asthma prevention and management. Future research should focus on identifying specific microbial signatures associated with asthma susceptibility and further investigate approaches like fecal microbiota transplantation. Understanding the role of gut microbiota in asthma pathogenesis can contribute to early detection and development of interventions to mitigate the risk of asthmatic pathogenesis in childhood.

Asthma, a chronic inflammatory disorder of the airways, affects approximately 10% of children globally, making it one of the most prevalent childhood chronic diseases.^1^ With a multifactorial etiology involving genetic and environmental factors, asthma poses a substantial global health burden.^2^ The diagnosis of asthma in children younger than 2 years of age poses a considerable challenge due to the absence of age-specific diagnostic tools.^3^ Additionally, accurately predicting the severity of asthma remains an ongoing obstacle in effective asthma management.^4^ The complex pathogenesis of asthma also contributes to the absence of well-defined therapeutic strategies, primarily stemming from an incomplete understanding of the underlying mechanisms.^5^

Recent studies have shed light on the interplay between the gut microbiota and the innate and adaptive immune systems, revealing their potential as crucial determinants in the etiology of asthma.^6^^,^^7^ There is a well-established relationship between the trillions of microbes that inhabit the human gut and organism-level health.^8^ Animal studies have provided compelling evidence that the gut microbiota and its metabolites play an influential role in the development of allergy disorders, particularly in infancy.^9^, ^10^, ^11^ Furthermore, microbial colonization of the gut in critical early periods of life plays an instrumental role in the education and training of the host immune system.^12^

Changes in this microbial composition, termed dysbiosis, has been linked to diseases of the respiratory tract, specifically through the critical cross talk between gut microbiota and lungs, also known as the gut–lung axis (GLA).^13^^,^^14^ The GLA is an organ-level interaction between the gut and lungs, allowing microbial metabolites and bioactive ligands to enter circulation and affect the host immune system.^15^ Higher microbial diversity has been linked to protective effects against the pathogenesis of childhood allergic diseases.^16^^,^^17^ Microbial colonization of infant mucosal surfaces, including the gastrointestinal tract, commences at birth.^18^ The mode of infant delivery itself has been implicated in the disruption of microbial colonization patterns. For instance, caesarean section births have been associated with higher levels of opportunistic nosocomial pathogens, including *Enterococcus, Enterobacter,* and *Klebsiella* species, in the infant gut microbiota.^19^ Gut microbial changes associated with caesarean section births have also been associated with an increased risk of asthma.^20^^,^^21^ In fact, a recent study by Lee-Sarwar and colleagues^22^ also showed an association between maternal prenatal gut microbiome, and the subsequent metabolome in infants. Specifically, the maternal microbial taxa *Erysipelatoclostridium, Terrisporobacter, Clostridia* UCG-014, *Clostridia* UBA1819, and *Lactobacillus* were found to be associated with transient asthma in infants, while *Faecalitalea* and *Prevotella* were found to be associated with active asthma in infants, as opposed to no asthma.

Other environmental factors also play a role in the pathogenesis of asthma as a result of their immune-modulating effects through targeting the gut microbiome. Lehtimäki and colleagues^23^ recently demonstrated that infants raised in urban areas had a higher risk of developing asthma and aeroallergen sensitivity. Infants from urban and rural areas had different gut microbiotas in terms of composition, and the authors postulated that the microbiome changes brought on by urbanization may increase the likelihood of asthma and atopic characteristics, most likely through interactions with the nascent immune system.

As a result of the demonstrated association between early life gut microbiota and the likelihood of childhood asthma, it is important to conduct studies that thoroughly analyze these patterns. A deeper understanding of the pathogenesis of asthma can aid in the early detection of risk factors and development of potential interventions that could lower the risk of pathogenesis.

One such area of intervention might be analysis of newborns’ gut microbiota. Gut microbiota is typically determined by collecting and analyzing stool samples—a noninvasive way to access and study the microbial communities residing in the gastrointestinal tract by measurement of their diversity.

Diversity is a fundamental aspect of microbial communities, encompassing both the number of different taxa detected within a sample and the differences in composition between samples. Alpha diversity measures the number of taxa detected per sample, providing insights into the richness and evenness of the microbial community within a specific sample.^24^ It serves as a quantitative metric to evaluate the species diversity within a given environment or biological system.^24^ Beta diversity, in contrast, focuses on the differences in composition between samples, allowing for the comparison of microbial community structures across different samples or environments.^25^ By assessing beta diversity, researchers can identify distinct microbial community patterns and discern specific relative abundances of bacteria and/or fungi at different taxonomic levels.^26^ These nuanced comparisons shed light on the variations in community structure and highlight the potential ecologic or functional implications of specific taxa within microbial ecosystems.^27^

The amplicon approach is a widely used method for analyzing stool samples to study the gut microbiome using 16S rRNA sequencing. In this approach, specific regions of the 16S rRNA gene are amplified, typically the V3-V4 or V4 region, using PCR with primers targeting the bacterial domain. This allows for targeted sequencing of the microbial community present in the sample.^28^ To determine the alpha diversity of the gut microbiome, amplicon sequencing data can be used to calculate various diversity metrics within a single sample. These metrics include observed species, Chao1, Shannon index, and Simpson index. They provide information about the richness (number of different taxa) and evenness (relative abundance distribution) of the microbial community within the sample.^28^ In addition to alpha diversity, the amplicon approach enables the assessment of beta diversity using various methods such as Bray-Curtis dissimilarity, weighted or unweighted UniFrac distances, and Jaccard index. These metrics quantify the dissimilarities in microbial community structure between samples, allowing for comparisons of the gut microbiome across individuals or experimental groups.^29^ By utilizing the amplicon approach with 16S rRNA sequencing, we can assess both the alpha and beta diversities of the gut microbiome, providing valuable insight into the composition and structure of microbial communities in stool samples.

A previous systematic review by Alcazar and colleagues^30^ in 2022 assessed the association between gut microbiota during infancy (measured from 0 to 1 year) and childhood respiratory diseases. Study inclusion was restricted to using genomic sequencing for measurement of gut microbiota during the first year of life. Because the stool sample analysis was restricted to having been measured within 1 year of life, the authors were unable to comment on the composition and diversity variability in the gut microbiota of children who went on to develop asthma and those who have an existing diagnosis of asthma. Furthermore, the systematic review included research published to April 27, 2021. As a result of emerging interest in this field, several primary studies have since been published. To our knowledge, this is the first systematic review to be conducted since 2021 to update this area’s current knowledge base.

In the present study, we systematically reviewed existing literature to examine the association between gut microbiota composition, as measured by 16S rRNA sequencing, during infancy and childhood and the subsequent development of atopic wheeze and/or asthma during childhood.

## Methods

A systematic literature review following the Preferred Reporting Items for Systematic Reviews and Meta-analysis (PRISMA) guidelines was conducted.

### Search strategy

A comprehensive search in 5 electronic databases (Medline [Ovid interface], Embase, Web of Science, Scopus, and Cochrane) was conducted for articles published in English from January 1, 2010, to May 1, 2023. The start year was chosen to ensure inclusion of all studies using genomic sequencing for gut microbiota characterization. The search terms used various combinations of the following terminology: “gastrointestinal microbiota,” “intestine,” “microbiota,” “respiratory disease,” and “asthma.” An information specialist was consulted to refine the search strategy, incorporating MeSH terms and keywords based on the inclusion of key studies. Additional studies were also identified by reviewing the references of relevant systematic reviews.

### Inclusion and exclusion criteria

Our criteria included original peer-reviewed research in humans, where child gut microbiota was the exposure, measured by genomic sequencing, with an outcome of asthma and/or wheezing.

### Preliminary screening strategy and data extraction

The first reviewer examined the titles, abstracts, and full text of the selected articles. A second reviewer evaluated 10% of the studies at each screening stage, and the results were compared to ensure agreement. The first reviewer performed data extraction. To ensure accurate reporting and reduce reviewer bias, a panel of secondary reviewers independently extracted information from all included studies. In cases of disagreement, a third reviewer was involved for resolution.

### Data synthesis strategy

The main results of all included studies were extracted and reported, which included differences in microbiota diversity, relative abundance of bacteria or fungi taxa (at the species, genus, or family level), and any measure of association in relation to diversity or abundance.

### Study quality assessment strategy

To evaluate the quality of included studies and provide context for interpreting the findings, 2 reviewers independently conducted a critical appraisal based on the Newcastle-Ottawa scale, a quality assessment tool that considers biases and limitations in study design. These results were translated into ratings of good, fair, or poor (see Tables E2-E5 in this article’s Online Repository at www.jaci-global.org) according to thresholds aligned with the standards set by the Agency for Health Research and Quality. In cases where studies included multiple analyses addressing different research questions, the quality assessments were applied only to the analyses relevant to this systematic review.

## Results

**Fig 1:**
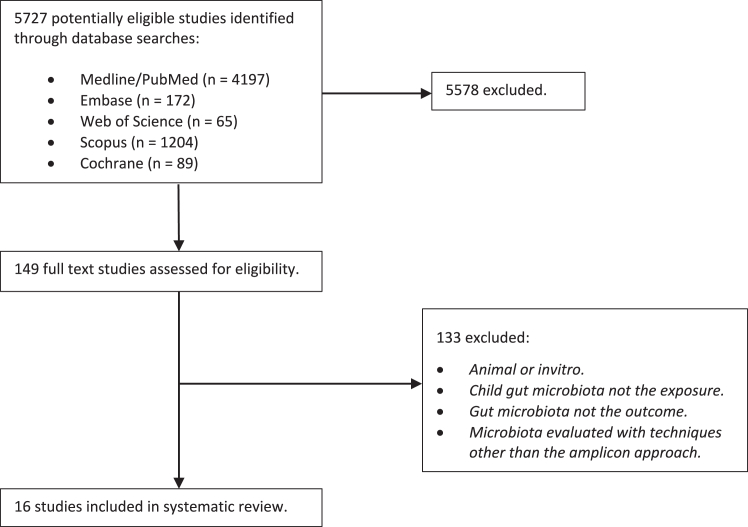
Study inclusion and exclusion process.

A total of 5727 titles and abstracts were reviewed and 5711 studies excluded. Inclusion criteria included peer-reviewed, original research in children where child gut microbiota was the exposure, as measured by 16S rRNA sequencing, and asthma and/or wheezing the outcome. The reasons for exclusion included nonprimary research, animal studies, *in vitro* studies, or studies in which probiotics were administered, or where the exposure or outcome did not align with the research question. After conducting a full-text review of the remaining 149 studies, 16 studies were deemed eligible for inclusion (Fig 1). Important findings from these studies are summarized in Tables I and II.

**Table I:** Study results stratified by age at stool sample collection and relative abundance of bacteria and fungal taxa in asthma versus no asthma

Study	Year	Age at gut microbiota determination	Age at asthma outcome determination	Time between gut microbiota assessment and asthma assessment	Alpha diversity	Relative abundance of bacterial taxa in children with asthma vs no asthma∗
Arrieta^42^	2015	• 3 months • 1 year	1 year (atopic wheeze) 3 years (asthma)	33 months/24 months	No difference	• At 3 months: Lower *Veillonella, Lachnospira, Rothia, Faecalibacterium* • At 1 year: Lower *Faecalibacterium, Lachnospira, Rothia, Veillonella, Peptostreptococcus*
Stiemsma^41^	2016	• 1 month • 1 year	4 years (asthma)	47 months/36 months	No difference	• Lower *Lachnospira* • Higher *Clostridium neonatale*
Fujimura^39^	2016	• 1 month • 6 months	4 years (high risk of asthma)	47 months/42 months	Not reported	• (Bacteria) Lower *Bifidobacterium, Lactobacillus, Faecalibacterium, Akkermansia* • (Fungi) Lower *Malassezia;* higher *Candida, Rhodotorula*
Stokholm^31^	2018	• 1 week • 1 month • 1 year	5 years (asthma)	60 months/59 months/48 months	No difference	• Lower *Roseburia, Alistipes, Flavonifractor* • Higher *Veillonella*
Arrieta^42^	2018	• 3 months	5 years (atopic wheeze)	57 months	No difference	• (Bacteria) Lower *Bifidobacterium;* higher *Streptococcus, Veillonella* • (Fungi) Higher *Pichia kudriavzevii*
Lee-Sarwar^32^	2019	• 3 years	3 years (asthma)	36 months	No difference	• Higher Christensenellaceae
Chiu^44^	2019	• 4-7 years with asthma, and healthy controls (n = 58)	4-7 years (asthma)	0 months (assessed in children with existing asthma + controls)	Not reported	• (Phylum) Lower Firmicutes • (Species) Lower *Faecalibacterium, Anaerostipes, Eubacterium, Roseburia;* higher *Clostridium* • (Genera) Higher *Escherichia, Alistipes, Bilophila, Enterococcus* • In healthy controls: Higher *Faecalibacterium, Roseburia*
Bannier^45^	2019	• 2-3 years with wheezing	2-3 years (wheeze)	Approximation: between 48 and 24 months	No difference	• Lower *Collinsella, Dorea* • Higher *Gemmiger, Escherichia*
Galazzo^33^	2020	• 5 weeks • 3.3 months • 5.3 months • 7.8 months		Approximation: between 65 and 131 months	No difference	• Lower *Lachnobacterium, Lachnospira, Dialister*
Patrick^34^	2020	• 3 months • 1 year	5 years (asthma)	60 months	Decreased alpha diversity	• Lower *Faecalibacterium prausnitzii, Ruminococcus bromii,* (Family) Rikenellaceae • (Genus) Higher *Dialister*
Depner^35^	2020	• 2 months • 1 year	6 years (asthma)	70 months/60 months	Decreased alpha diversity	• At age 2 months: Lower *Bacteroides, Parabacteroides;* higher *Enterococcus* • At age 1 year: Lower *Roseburia, Ruminococcus, Faecalibacterium*
Boutin^36^	2020	• 3 months	1 year (recurrent wheeze and atopic wheeze)	9 months	Increased alpha diversity at 3 months was protective of recurrent and atopic wheeze	• Children with recurrent wheeze vs healthy children: Lower *Faecalibacterium, Lachnospira, Coprococcus, Oscillospira* • Children with atopic wheeze vs healthy children: Lower *Faecalibacterium, Lachnospira, Coprococcus, Roseburia, Blautia, Parabacteroides, Ruminococcus*
Boutin^37^	2021	• 3 months • 1 year	5 years (asthma)	57 months/48 months	*Three-month stool sample:* Increased alpha diversity associated with inhalant atopy. *One-year sample:* Decreased alpha diversity associated with inhalant atopy.	• *Saccharomyces cerevisiae* (ASV1) found to be indicator from infants who developed inhalant atopy at age 5 years • At 3 months: Lower *Malassezia;* higher *Rhodotorula,* non-*albicans Candida* • At 1 year: Lower *Debaryomyces, Saccharomyces, Candida;* higher allergy-associated fungi such as *Alternaria*
Hsieh^40^	2021	• Children aged 9-17 years	9-17 years (asthma)	0 months (assessed in children with existing asthma + controls)	No difference	• No difference reported
Lee-Sarwar^38^	2022	• Children with asthma at 3 years	3 years (high risk of asthma; recurrent wheeze)	0 months	No difference	• Lower *Holdemanella, Senegalimossilia, Colidextribacter, Eubacterium fissicatena* group; (Family) Lachnospiraceae (Lachnospiraceae CAG-56), Oscillospiraceae (Oscillospiraceae UCG-003, Oscillospiraceae UCG-005), Ruminococceae (Ruminococceae CAG-352) • Higher *Veillonella, Roseburia, Parasuterella, Ruminococcus* (*Ruminococcus gouvreauii* group), *Eubacterium (Eubacterium siraeum* group), *Clostridium* (*Clostridium* sensu stricto 1); (Class) Clostridia UCG-014; (Family) Anaerovoracaceae Family XIII AD1 group
Lee-Sarwar^22^	2023	• 3-6 months • 1 year • 3 years	3 years (high risk of asthma; recurrent wheeze) 6 years (asthma)	Approximation: between 36 and 69 months	No difference	• Lower *Staphylococcus, Bacteroides*

∗ Reported at genus level unless otherwise stated.

**Table II:** Summary of trends of higher or lower relative abundance of bacterial or fungal taxa in asthma versus no asthma

Relative abundance of microbes associated with asthmatic outcomes	Microbe	References
Lower	Bacteria	
	*Bifidobacterium*	40, 43
	*Faecalibacterium*	34, 35, 36, 40, 43, 45
	*Lachnospira*	33, 36, 39, 42, 43
	*Roseburia*	31, 35, 36, 45
	*Ruminococcus*	34, 36, 39
	*Bacteroides*	35, 38
	Fungi	
	*Malassezia*	37, 40
Higher	Bacteria	
	*Veillonella*	31, 39, 43
	*Clostridium*	42, 45
	*Escherichia*	45, 46
	Fungi	
	*Rhodotorula*	37, 40

Of the 16 studies included, 10 were cohort studies,^22^^,^^31^, ^32^, ^33^, ^34^, ^35^, ^36^, ^37^, ^38^, ^39^ 1 was an observational study,^40^ 3 were nested case–control studies,^41^, ^42^, ^43^ and 2 were prospective case–control studies.^44^^,^^45^

All included studies used 16S rRNA sequencing from V3 and/or V4 regions to analyze the bacterial composition. Three studies^33^^,^^41^^,^^43^ used the V3 region, and 10 studies^22^^,^^31^^,^^34^, ^35^, ^36^, ^37^, ^38^, ^39^, ^40^^,^^42^ used the V4 region. Two studies^44^^,^^45^ used both V3 and V4 regions. Eight studies^22^^,^^31^^,^^32^^,^^35^^,^^37^, ^38^, ^39^^,^^42^ used the Illumina MiSeq sequencing platform, and 3 studies^34^^,^^43^^,^^44^ used Illumina HiSeq. Five studies^22^^,^^32^^,^^38^^,^^40^^,^^42^ used the SILVA database,^46^ while 9 studies^31^^,^^34^, ^35^, ^36^, ^37^^,^^39^^,^^41^, ^42^, ^43^ used the Greengenes reference database. Four studies^35^^,^^37^^,^^39^^,^^42^ assessed the fungal microbiome. One study used the fungal internal transcribed spacer region 1 UNITE dynamic database,^35^ another used 18S rRNA V4,^42^ the third used ITS-2 rRNA gene sequencing, quantitative PCR–based total fungus load,^37^ and the last used fungal internal transcribed spacer region 2 UNITE database V6.^39^

Sample sizes varied between the 16 included studies. The smallest sample size was 58 subjects,^44^ while the largest sample size was 917 individuals,^34^ yielding a mean of 487.5 individuals. Stool samples were collected at various ages depending on the study. The earliest stool sample collection took place postpartum, from 1-month-olds (n = 306),^33^ and the oldest participants had their stool samples taken between ages 9 and 17 years (n = 80).^40^

In most studies, asthmatic status was evaluated several years after the stool samples were gathered, and ages varied from neonates to 17 years. Of the 16 studies, 3 studies included stool samples from infants who were 1 month old.^31^^,^^39^^,^^41^ Six studies included stool samples from infants at 3 months old.^22^^,^^34^^,^^36^^,^^37^^,^^42^^,^^43^ Seven studies included stool samples from children at 1 year of age.^22^^,^^31^^,^^34^^,^^35^^,^^37^^,^^41^^,^^43^ Ages 1 and 3 months were the most explored.

The included studies used various methods^47^ of evaluating the presence of asthma and/or respiratory disease status such as atopic wheeze in the study group. All studies used clinical assessment to evaluate outcomes. Two studies additionally used skin prick test response to assess atopic wheeze.^42^^,^^43^ Nine studies^22^^,^^32^^,^^34^^,^^36^^,^^38^^,^^40^^,^^41^^,^^43^^,^^45^ mentioned the use of questionnaires filled out by the parents to assess outcomes, whereas 3 studies^34^^,^^41^^,^^45^ mentioned the used of ISAAC (International Study of Asthma and Allergies in Childhood) questionnaires. One study additionally used the asthma predictive index (aka API) to predict the incidence of active asthma between 6 and 13 years.^43^ Furthermore, some studies subdivided asthma into several categories. For example, one study compared early, active, and transient asthma.^22^ Another study subdivided asthma into moderate–severe and well-controlled asthma.^40^

Most studies had some adjustment for confounders, such as breast-feeding, presence of older siblings, or delivery method. Some studies used questionnaires to document confounders. For example, in one study, questionnaires were used to gather information about environmental exposures, psychosocial stresses, nutrition, and general health.^43^

Thirteen of 16 studies mentioned assessment of alpha diversity regarding asthma and/or wheeze outcome. The 3 remaining studies did not mention alpha diversity assessment.^32^^,^^39^^,^^44^ Eight studies reported no significant difference in alpha diversity.^22^^,^^31^^,^^38^^,^^40^, ^41^, ^42^, ^43^^,^^45^ One study showed that an increased alpha diversity had protective effects against the development of asthma at 1 year of age (*P* = .046).^34^ Another study assessed alpha diversity using principal component analysis (PCA).^35^ It found that at 2 months, PCA axis showed a protective effect (*P* = .024) that correlated positively with abundance of *Bacteroides* and *Parabacteroides* and negatively with *Enterococcus*. In this study, the estimated microbiome age (EMA) was strongly correlated with PCA at month 12 (*P* = .75) and alpha diversity (*P* = .70) but not with PCA at 2 months. One study showed that increased alpha diversity at age 3 months was protective for recurrent wheeze (*P* = .007) and atopic wheeze (*P* = .016).^36^ Another study showed that at 3 months, increased alpha diversity was associated with inhalant atopy (*P* = .21). Conversely, the same study showed that at 1 year of age, a decreased alpha diversity was associated with inhalant atopy.^37^

In 8 of 16 studies mentioned, beta diversity was assessed regarding asthma and/or wheeze outcome. The 8 remaining studies^32^, ^33^, ^34^, ^35^, ^36^^,^^41^^,^^43^^,^^44^ did not mention beta diversity. In 4 of the studies that mentioned beta diversity, there was no significant difference reported.^38^^,^^40^^,^^42^^,^^45^ One study reported that bacterial (PERMANOVA; *R*^2^ = 0.09, *P* < .001) and fungal Bray-Curtis; PERMANOVA, *R*^2^ = 0.037, *P* = .068) beta diversity differed between clusters in children with high risk of asthma compared to those with low risk.^39^ Another study^31^ found no difference in beta diversity at 1 week and 1 month but found a difference in beta diversity in 1-year-olds with asthma, contrary to asthma-free 1-year-olds (PERMANOVA; *F* = 3.4, *R*^2^ = 0.6%, *P* = .003). With the collection of 3-month stool samples, another study determined significant differences in beta diversity associated with sensitization to inhalant allergies at 5 years of age.^37^ However, no differences were found at 1 year of age. Finally, one study reported beta diversity associated with active asthma in 3-year-olds (PERMANOVA; *F* = 2.50, *P* = .004).^22^

Fourteen of the 16 included studies reported microbial diversity and relative abundance. Only one study reported no difference in microbial diversity in children with asthma compared to control subjects.^40^ One study focused entirely on fungi.^37^ In the remaining studies, microbial diversity varied; however, lower abundance in *Faecalibacterium* at 3 months and 1 year was reported in 6 studies.^34^, ^35^, ^36^^,^^39^^,^^43^^,^^44^ Lower abundance in *Lachnospira* was reported in 5 studies^33^^,^^36^^,^^38^^,^^41^^,^^43^ at 3 months and in one study^38^ at 3 years. Lower abundance in *Bifidobacterium* was found in 2 studies.^39^^,^^42^ Decrease in relative abundance of *Roseburia* in 4 studies,^31^^,^^35^^,^^36^^,^^44^ *Ruminococcus* in 3 studies,^34^^,^^36^^,^^38^ and *Bacteroides* in 2 studies^22^^,^^35^ was found to be associated with asthmatic outcomes. Higher relative abundances of *Veillonella*,^31^^,^^38^^,^^42^ *Clostridium,*^41^^,^^44^ and *Escherichia*^44^^,^^45^ were found to be associated with asthma and asthmatic outcomes at ages 1 to 7 years.

Four studies investigated the association of fungal composition and asthma.^35^^,^^37^^,^^39^^,^^42^ One study reported lower abundance (*P* < .05) of *Malassezia* and higher abundance of *Candida* and *Rhodotorula* in children with high risk of asthma compared to those at low risk.^39^ In one study, asthmatic individuals had a difference in EMA at 2 months of age and had lower EMA at age 1 year than children without asthma.^35^ In another study, asthmatic individuals had a difference in EMA at age 2 months and lower EMA at age 1 year than children without asthma.^35^ One study collected samples at both 3 months and 1 year, and showed that *Saccharomyces cerevisiae* was found to be an indicator in infants for development of inhalant atopy at age 5 years (stat = 0.882; *P* = .005), atopy at age 5 years (stat = 0.879; *P* = .005), or asthma at age 5 years (stat = 0.892; *P* = .005).^37^ This study also reported a lower abundance of *Malassezia* and a greater abundance of *Rhodotorula*, non-*albicans Candida* at 3 months; lower abundance of *Debaryomyces, Saccharomyces,* and *Candida,* and a higher abundance of allergy-associated fungi such as *Alternaria* at 1 year of age in children who developed inhalant atopy compared to a control group (*P* < .05).

## Discussion

Results of this systematic review found a trend showing that a higher alpha diversity of bacteria is protective against asthmatic outcomes in children.^34^, ^35^, ^36^ Previous studies have also shown a reduced bacterial diversity in airways of individuals with asthma, especially those with higher eosinophilic inflammation.^48^^,^^49^ It is possible that the GLA plays a significant role in the microbial composition dynamics of both the airways and the gut. However, most studies included in this systematic review reported alpha diversity as not having a significant difference on asthmatic outcomes.^22^^,^^31^, ^32^, ^33^^,^^38^^,^^40^, ^41^, ^42^, ^43^^,^^45^ One study reported greater alpha diversity of fungi at 3 months, and lower alpha diversity of fungi at 1 year, to be associated with asthmatic outcomes.^37^

Results trended toward lower relative abundances of the bacteria *Bifidobacterium,*^39^^,^^42^ *Faecalibacterium,*^34^, ^35^, ^36^^,^^39^^,^^43^^,^^44^ *Lachnospira,*^33^^,^^36^^,^^38^^,^^41^^,^^43^ *Roseburia,*^31^^,^^35^^,^^36^^,^^44^ *Ruminococcus,*^34^^,^^36^^,^^38^ and *Bacteroides*^22^^,^^35^ being associated with asthma or atopic wheeze between ages 1 and 7 years. In contrast, higher relative abundances of *Veillonella,*^31^^,^^38^^,^^42^ *Clostridium,*^41^^,^^44^ and *Escherichia*^44^^,^^45^ were found to be associated with asthma and asthmatic outcomes at ages 1 to 7 years. Lower relative abundance of the fungi *Malassezia* and higher relative abundance of *Rhodotorula* in infancy were also found to be associated with asthmatic outcomes in children aged between 4 and 5 years.^37^^,^^39^

A previous systematic review by Alcazar and colleagues^30^ explored the association between infant gut microbiota and childhood respiratory diseases, such as wheezing, asthma, and respiratory infections. It included 11 studies, with the most recent being from 2020. Nine of them were included in our systematic review,^31^^,^^33^, ^34^, ^35^, ^36^^,^^39^^,^^41^, ^42^, ^43^ but 2 were excluded because they tested respiratory infections as an outcome rather than asthma or atopic wheeze. The authors concluded that there was evidence linking childhood respiratory illnesses to low alpha diversity and relative abundance of the specific gut-commensal bacteria genera *Bifidobacterium, Faecalibacterium, Ruminococcus,* and *Roseburia*. Three of the studies included in their review also reported findings on fungi composition, although all studies showed varying fungal associations with asthmatic outcomes.^35^^,^^39^^,^^42^ In addition to these 3 studies, a study by Boutin et al^37^ was included in our review that also assessed fungal composition. Inclusion of this single additional study enabled us to find at least 2 studies that show the association of lowered relative abundance of *Malassezia* and higher relative abundance of *Rhodotorula* with asthmatic outcomes.^37^^,^^39^

Caution must be practiced before absolute generalizations can be made on the associations found between specific microbiota and their impact on asthmatic outcomes as a result of the heterogenous sample sizes, definition of outcomes, follow-up times, bioinformatic and statistical approaches, and reference databases utilized for bacterial taxonomic classification. Additionally, although an exhaustive search was conducted, there is a chance we missed some smaller studies when we executed our search.

Eleven of 16 studies collected stool samples at the age of 1 year or younger. Seven of 16 studies collected stool samples at 3 months of age, with 5 before the age of 3 months.^22^^,^^31^^,^^34^, ^35^, ^36^, ^37^^,^^39^^,^^41^, ^42^, ^43^ Similarly, 7 studies also collected stool samples at age 1 year.^22^^,^^31^^,^^34^^,^^35^^,^^37^^,^^41^^,^^43^

Four studies collected stool samples at 3 years of age.^22^^,^^32^^,^^38^^,^^45^ Only 2 studies collected stool samples at an age greater than 3 years: one between ages 4 and 7 years and another between ages 9 and 17 years.^40^^,^^44^ Most asthma diagnoses occurred around 3 years and beyond. Evidently there is a greater percentage of asthma diagnoses made via stool samples taken at a later age.^40^^,^^44^

Six of the 11 studies that collected stool at 1 year of age or younger showed no difference in alpha diversity.^22^^,^^31^^,^^33^^,^^41^, ^42^, ^43^ However, 2 studies displayed an increase in alpha diversity at 3 months of age.^34^^,^^35^ In contrast, 3 studies that collected stool at 1 year old showed a decrease in alpha diversity.^34^^,^^35^^,^^37^ This further solidifies the theory that a diverse gut microbiome at 3 months of age is protective against the future development of asthma. Four studies of children with stool collected at 3 months old showed a decrease in *Lachnospira*.^33^^,^^36^^,^^38^^,^^43^ Greater *Escherichia* abundance was seen in children where the stool sample was taken from 2 to 7 years of age.^44^^,^^45^ Last, children who had samples taken between the ages of 9 and 17 years showed no significant difference in gut microbiota than the control, as well as absence of alpha and beta diversity.^40^

Any comparison between studies that analyzed stool samples within the first year of life (0-1 years), as opposed to those that tested stool samples from older children (above 3 years) should be interpreted carefully. While acknowledging this caveat, this systematic review did reveal discernible patterns in the gut microbiota composition of younger children exhibiting asthmatic outcomes compared to older children with asthmatic outcomes. In both age groups, elevated levels of *Clostridium, Enterococcus,* and *Veillonella* were observed, while diminished levels of *Faecalibacterium,*^34^, ^35^, ^36^^,^^39^^,^^43^^,^^44^ *Lachnospira,*^33^^,^^36^^,^^38^^,^^41^^,^^43^ and *Ruminococcus*^34^^,^^36^^,^^38^ were noted. Regarding distinctions between the age groups, children aged 0 to 1 year manifested lower concentrations of *Bacteroides,*^22^^,^^35^ *Bifidobacterium,*^39^^,^^42^ and *Roseburia*.^31^^,^^36^^,^^44^ Conversely, children aged 3 to 17 years exhibited higher levels of *Escherichia*.^44^^,^^45^ Conclusions about studies that analyzed stool samples collected after 1 year should be treated with caution because administration of inhaled corticosteroids and β-agonists can also influence gut microbes, resulting in less-diverse bacteria.

### Microbial associations

#### Bacteria

According to the results of this systematic review, a significant decrease in levels of *Bifidobacterium* was observed in children with asthma or atopic wheeze. *Bifidobacterium* is a predominant genus of bacteria in the infant gut and has been demonstrated to play a crucial role in immune modulation and immune system development by stimulating the maturation of gut-associated lymphoid tissue and enhancing the production of regulatory T cells.^50^ Furthermore, *Bifidobacterium* has been shown to produce an exopolysaccharide that is connected with the cell surface, the presence of which reduced immune response from host B cells.^51^ Several studies have explored the effects of supplementing gut microbiota with *Bifidobacterium* in animal models to evaluate its impact on atopic wheeze and asthmatic outcomes. One murine model of chronic allergic asthma demonstrated the effectiveness of *Bifidobacterium breve* treatment as being comparable to budesonide in reducing inflammation.^52^ Another study demonstrated strong anti-inflammatory properties of *Bifidobacterium breve* in conjunction with oligosaccharides in a murine ovalbumin-induced chronic asthma model.^53^ Results of this combined treatment demonstrated repressed pulmonary airway inflammation, decreased T cell-activation and mast cell degranulation, and prevented airway remodelling.

*Faecalibacterium, Lachnospira* and *Roseburia* are commensal bacteria found in the infant gut that have been identified as having an important role in immune modulation through their production of butyrate.^54^ Butyrate is a short-chain fatty acid known for possessing anti-inflammatory properties. It has been shown to promote production of anti-inflammatory cytokines such as IL-10.^55^^,^^56^ Animal studies have also shown its involvement in promotion of differentiation and function of regulatory T cells and dendritic cells, which play a crucial role in maintaining immune tolerance through the prevention of excessive immune responses.^43^^,^^57^ A decreased level of fecal butyrate is associated with greater levels of IgE and risk of early childhood asthma.^44^ In contrast, there is a negative correlation seen with increased levels of *Clostridium* and butyrate levels. In fact, *Clostridium* suppresses butyrate production.^44^ Studies that have explored the use of butyrate as treatment in a mouse model of allergic asthma have shown reduced airway hyperreactivity and lung inflammation as well as improved respiratory function.^58^^,^^59^ Further studies that test the therapeutic use of butyrate-producing bacteria *Faecalibacterium, Lachnospira* and *Roseburia* in animal models could be useful in determining probiotic strategies to provide prophylaxis against asthmatic risk and pathogenesis.

#### Fungi

Fungi play an important role in the developing immune system.^60^ Asthma is well known to be triggered by inhaled fungi, and individuals who are sensitized to fungi have higher rates of developing asthma compared to those who are sensitized to other allergens.^61^

In the present systematic review, lower relative abundance of the fungi *Malassezia* was found to be associated with increased asthmatic outcomes. Although there are several studies that have demonstrated the importance of skin microbiota, and specifically *Malassezia*, in atopic dermatitis, only few have investigated the serology of *Malassezia* IgE in asthmatic patients.^62^ However, atopic dermatitis is itself linked to the increased incidence of atopic respiratory illnesses, including asthma, and therefore careful analysis of these studies might provide insights into the role *Malassezia* may play in allergy.^63^ Incidentally, Fujimura et al^39^ reported *Malassezia* as being “characteristically enriched” in newborn gut microbiota. Further studies focused on its biochemical role in immune modulation may be required to understand how its dysbiosis in the gut might influence asthma development.

In contrast, *Rhodotorula* was found to be increased in children at high risk of developing asthma. Previously, airway microbiota of individuals with eosinophilic asthma has been found to be enriched with *Rhodotorula*.^64^ It would be interesting to compare results of studies that determine both airway and gut microbiota in order to determine the extent of GLA interaction and the role it may play in the regulation of asthmatic outcomes.

*Candida* was another fungus found to be associated with asthma in children. While Fujimura et al^39^ found higher levels of gut *Candida* at 1 and 6 months to be associated with asthma at 4 years, Boutin et al^37^ found higher levels of non-*albicans Candida* at 3 months and lower levels of *Candida* at 1 year to be associated with asthma at age 5 years. While these results are contradictory, previously, *Candida albicans*–specific IgE sensitization has been shown to be strongly correlated with persistent serious illness in asthma.^65^ *Candida* in the gut produces prostaglandin E_2_, which can travel to the lungs and induce lung M2 macrophage polarization, leading to allergic airway inflammation.^15^ Further studies focusing on determining the association of *Candida* dysbiosis in the gut to asthmatic outcomes are required.

### Clinical implications

The association between gut microbiota composition and asthma pathogenesis has significant clinical implications. Recent studies have even demonstrated that early asthma is linked to maternal fecal beta diversity during pregnancy (PERMANOVA; *F* = 1.65, *P* = .05).^22^ In the future, early screening and risk assessment of gut microbiota composition from both infants and pregnant mothers may help determine microbial signatures associated with asthma susceptibility, therefore helping identify at-risk individuals.

Within the scope of this systematic review, it is important to acknowledge a notable limitation inherent in the analyzed studies. A discernible gap emerges as the studies did not systematically consider obesity as a confounding factor when evaluating asthmatic status. This oversight gains significance considering the findings by Michalovich et al,^66^ which emphasizes the influential role of obesity in restructuring microbiota composition and amplifying perturbed microbiome–immune interactions among asthma patients. The absence of obesity as a controlled variable introduces a potential confounding influence, thereby warranting a cautious interpretation of the identified associations between gut microbiota dysbiosis and childhood asthma. Furthermore, the investigation by Michalovich et al offers compelling insights into a prospective avenue for therapeutic intervention. Specifically, it highlights a potential nonredundant role for *Akkermansia muciniphila* in individuals exhibiting a severe asthma phenotype. The current systematic review did not identify diminished levels of *A muciniphila* in children with asthmatic outcomes. Nevertheless, the observed inverse correlation between the severity of asthma and fecal *A muciniphila* levels, coupled with demonstrated alleviation of airway hyperreactivity and inflammation in murine models after *A muciniphila* administration, underscores the need for sustained research in this direction. Such investigations could hold promise for uncovering personalized therapeutic approaches to mitigate repercussions of severe asthma in pediatric populations.

Treatment strategies against asthma risk and severity may also potentially include possible measures to restore the gut flora to normal levels. Dietary factors play a pivotal part in shaping the composition of the microbiota, and nutritional interventions aimed at modifying the gut microbiota should thus be considered in asthma management. A notable limitation of this proposition stems from an inherent constraint in the methodologies used across the studies we included in this systematic review. Specifically, there is a lack of consideration of dietary patterns among children with asthma in contrast to their nonasthmatic counterparts. Nevertheless, it is pertinent to acknowledge that interventions involving high-fiber diets have been implemented in mouse models to investigate asthmatic outcomes, although they have yielded conflicting results.^67^^,^^68^ For instance, Wen et al^67^ examined the effects of a high-cellulose diet in a mouse model of asthma and found pathologic lung symptoms, changes in intestinal microbiota, and alterations in short-chain fatty acids in the mouse intestines. In mice with asthma, a diet high in cellulose was found to lessen lung inflammation and asthmatic symptoms. In contrast, Zhang et al^68^ found that an excessively high-fiber supplement had a promoting allergic effect rather than a protective one in allergic airway disease model mice. The high cellulose supplement increased nasal rubbing and sneezing, eosinophil inflammation, and goblet cell metaplasia in subepithelial mucosa, and it promoted T_H_2 skewing of the immune response as well as production of serum levels of ovalbumin-specific IgE. Further research in animal and human models can address the discrepancies and may suggestive an optimal level of fiber supplementation for protective effects.

Probiotic interventions have emerged as a potential therapeutic approach in modulating gut microbiota to mitigate asthmatic symptoms. Probiotics are live microorganisms that, when administered in adequate amounts, confer health benefits to the host. Various studies have investigated the efficacy of probiotics in asthma management, albeit with conflicting results.^69^, ^70^, ^71^ In a meta-analysis of randomized control trials conducted between 2009 and 2019, Wawryk-Gawda et al^71^ found postnatal probiotics supplementation ineffective in asthmatic risk reduction; however, in children who received prebiotics or synbiotics, some risk prevention factors were observed. In contrast, a meta-analysis of randomized controlled trials conducted by Uwaezuoke and colleagues^70^ targeting studies between 2017 and 2022 did find postnatal strain-specific probiotics to be effective in preventing allergies, and also found specific strains to be more effective in improving asthmatic outcomes. A systematic review by Colquitt et al^69^ focused on probiotic supplementation during pregnancy to determine risk reduction effects in asthmatic outcome of children. While findings were mixed, there was some evidence that prenatal probiotics are beneficial in children at high risk for developing allergies. To infer a more conclusive answer on the effectiveness of nutritional interventions, larger-scale research is necessary, with at-risk groups as the focal point of such investigations.

Fecal microbiota transplantation (FMT), which involves transferring microbiota from a healthy donor to a recipient, has demonstrated encouraging outcomes in the management of various gastrointestinal illnesses.^72^ In one study, bacteriotherapy activated a specific pathway involving the MyD88 protein and the ROR-γt protein in regulatory T cells. This helps protect against food allergies, which were lacking in food-allergic children and mice.^73^ Although its efficacy has not been studied properly in the context of asthma, early research points to a potential therapeutic benefit.^74^ A recent systematic review exploring the effects of FMT on prevention of allergies concluded that, while promising, further research is required in both animal and human models.^75^ Further research is thus warranted to investigate the long-term effects and optimal protocols for FMT in asthma treatment.

### Conclusion

The interplay between gut microbiota composition and childhood asthma is a topic of significant interest. This systematic review highlights the potential association between specific microbial taxa and asthmatic outcomes, suggesting that higher bacterial alpha diversity and certain beneficial bacteria may have protective effects, while lower abundances of specific bacteria and fungi may be associated with increased asthma risk. Because lower relative abundances of the bacteria *Bifidobacterium,*^39^^,^^42^ *Faecalibacterium,*^34^, ^35^, ^36^^,^^39^^,^^43^^,^^44^ *Lachnospira,*^33^^,^^36^^,^^38^^,^^41^^,^^43^ *Roseburia,*^31^^,^^35^^,^^36^^,^^44^ *Ruminococcus,*^34^^,^^36^^,^^38^ and *Bacteroides,*^22^^,^^35^ as well as the fungus *Malassezia,*^37^^,^^39^ were found to be associated with asthma or atopic wheeze in young children, these bacteria may be considered to play a beneficial role. These findings underscore the importance of early life gut microbiota in shaping the immune system and influencing asthma development. Further research is needed to identify microbial signatures associated with asthma susceptibility, allowing for early identification of at-risk individuals and development of targeted preventative and therapeutic interventions against asthma. Strategies such as dietary modifications, probiotic supplementation, and FMT hold promise and warrant further investigation. By unraveling the intricate relationship between gut microbiota and childhood asthma, we can pave the way for individualized approaches to asthma prevention and treatment.

## Disclosure statement

Supported by Uniwersytet Jagiellonski, UJ Collegium Medicum.

Disclosure of potential conflict of interest: The authors declare that they have no relevant conflicts of interest.
