# Supplementary material for: Link between gut microbiota dysbiosis and childhood asthma: Insights from a systematic review
Source: J Allergy Clin Immunol Glob. 2024 Jun 12;3(3):100289. doi: 10.1016/j.jacig.2024.100289 (PMC11298874; doi:10.1016/j.jacig.2024.100289)
Supplement: Supplementary data [file mmc1.docx]

Table 1. Summary of studies included in systematic review, including details about participants, exposure and outcome, relative abundances of microbial taxa as reported in each study, and quality of study.

| Study;  Microbiota determination Technique | Number of participants; age at stool sample | Asthma outcome; age at outcome | Time lapse between gut microbiota assessment and asthma assessment | Method of evaluation; outcome definition | Number of participants in each outcome group | Diversity and relative abundance of microbial taxa in children with asthma as compared to healthy subjects (reported at genus level, unless otherwise stated) | Study quality |
| --- | --- | --- | --- | --- | --- | --- | --- |
| Arrieta et al (2015)[44]; nested case- control study  16S rRNA sequencing, V3 region, Ilumina HiSeq, and Greengenes database (2006) | CHILD study cohort (n = 319); at 3 months at 1 year | Atopic wheeze; at age 1 year   Asthma; at age 3 years | 33 months/  24 months | Parent reported questionnaires, filled out at three months, six months and one year. Asthma Predictive Index (API) was used to predict incidence of active asthma between six and 13 years of age. Clinical data was collected by CHILD clinician during two- and three-year visits, which was used to determine clinical significance of the one-year phenotypes.   *(During first year of life)* **Wheeze:** Presence of wheeze with or without cold **Atopy:** positive prick test **Wheeze:** more than wheezing episode **Control individuals**: no asthma or atopy | Atopy (n = 87) 27% Wheeze (n = 136) 43% Atopy + wheeze (n = 22) 7% Asthma at age 3 (n = 19) 5.96% Control (n = 74) 23% | **In children with atopy and wheeze, as compared to control individuals** at 3 months: Lower *Faecalibacterium*, *Lachnospira*, *Rothia*, *Veillonella*, and *Peptostreptococcus* (p < 0.05) at 1 year: Lower *Oscillospira* (p = 0.03)  **Alpha diversity:** No significant difference | Poor |
| Stiemsma et al (2016)[42]; nested case-control study  16S rRNA sequencing, V3, Ilumina and Greengenes database (2006) | CHILD Study cohort (n = 268)  Newborns (n = 76); at 1 month at 1 year | Asthma; at age 4 years | 47 months/  36 months | Parent reported physician diagnosis asthmatic medication prescription and use by age of four years. At ages one, three and five years, ISAAC Questionnaire reports obtained from parent or legal guardian, and clinical assessment made by CHILD Study clinicians.  **Asthma:** physician diagnosis or confirmed asthmatic medication prescription and use. **Control:** absence of asthma diagnosis or medication prescription. | Asthma (n = 39) 51% Controls (n = 37) 49% | **Children with asthma as compared to control subjects** at age three months: (**Genera)** Lower *Clostridiales* (p = 0.035) and *Lachnospira* (p = 0·098); (**Species)** Higher *Clostridium neonatale* (p = 0·076); (**Family)** Higher *Clostridiaceae* (p = 0·005); (**Phylum)** Higher *Firmicutes* (p = 0·035)  At age one year: **Genera:** Higher *Rothia* (p = 0·003): **Family:** Higher *Lachnospiraceae* (p = 0·032)   **Alpha diversity:** No difference | Fair |
| Fujimara et al (2016)[40]; cohort study  16S rRNA sequencing, V4 region, Illumina MiSeq, Greengenes database (2013); Fungal internal transcribed spacer region 2, UNITE database V6 | Newborns (n = 279); 1 month (n = 111)  6 months (n = 168) | Asthma; at age 4 years | 47 months/  42 months | Parental interviews at age one month, six months, one year, two years and four years. Clinical study visits at age two years. Parent reported physician diagnosed Asthma by age four years. | Asthma (n = 39) 14% | **Children with high risk of asthma, as compared with low-risk asthma participants Bacterial taxa (p < 0.05);** Lower *Bifidobacterium, Lactobacillus, Faecalibacterium,* and *Akkermansia*  **Fungal taxa (p < 0.05):** Lower *Malassezia;* Higher *Candida* and *Rhodotorula*  **Alpha diversity:** Not reported **Beta-diversity (**Bacterial): (PERMANOVA; R2=0·09, p<0·001) and Beta-diversity (Fungal): (Bray–Curtis; PERMANOVA, R2=0·037, p=0·068) differed between clusters; | Fair |
| Stokholm et al (2018)[31]; cohort study  16S rRNA sequencing, V4 region, V4, Illumina Miseq, and Greengenes database (2013) | Newborns (n = 690); at 1 week at 1 month at 1 year | Asthma; at age 5 years; (n = 648) | 60 months/  59 months/  48 months | Pediatrician diagnosed asthma diagnosis. Clinical visits at ages one week, one, three, six, 12, 18, 24, 30, and 36 months, and yearly thereafter (including during acute respiratory episodes). Asthma diagnosis based on combination of five episodes of lung symptoms within six months, (where each episode lasted at least three consecutive days); Symptoms triggered by exercise, prolonged coughing at night, or persistent coughing unrelated to common colds. Use of β2-agonist for relief and with positive response to three months of inhaled steroids but had relapse upon discontinuation. | Asthma (n = 60) 9% | **Individuals with asthma, as compared to control subjects** At ages one week or one month: No differences  At one year: Lower *Roseburia* (p=0·042), *Alistipes* (p=0·002) and *Flavonifractor* (p=0·002); Higher *Veillonella* (p=0·035)   **Alpha-diversity:** No difference  **β-diversity:** At ages one week and one month: no difference; at one year in individuals with asthma vs individuals without asthma (PERMANOVA; F=3·4, R2=0·6%, p=0·003); | Good |
| Arrieta et al (2018)[43]; nested case-control study  16S rRNA sequencing, V4 region, Miseq Ilumina, Greengenes database (2006); 18S rRNA V4 (fungi), SILVA database (2013) | Newborns (n = 97);  at 3 months | Atopic wheeze; at 5 years | 57 months | **Atopic wheeze:** maternally reported wheeze in the previous 12 months and positive skin prick test response at age five years. **Controls**: random sample of children without history of wheeze or evidence of atopy at age five years. | Atopic wheeze (n = 27) 28% Control subjects (n = 70) 72% | **Children with atopic wheeze, as compared to control subjects** Lower *Bifidobacterium* (p<0·001)  Higher *Streptoccocus* (p=0·044) and *Veillonella* (p=0·031)  **Relative abundance of fungal taxa (genera):**  Higher *Pichia Kudriavzevii* (p<0·01)  **Alpha-diversity, Beta diversity (bacterial or fungal):** No difference | Good |
| Lee-Sarwar et al (2019)[47]; prospective cohort study  16S rRNA V4, Illumina MiSeq, SILVA database | Newborns VDAART (multi-site) participants (n = 806)  Stool at age 3 years (n = 361) | Asthma; by 3 years of age | 36 months | Parental report of physician diagnosis of asthma or occurrence of recurrent wheeze as determined by questionnaires administered postnatally, and every three months until child reached three years of age. | Asthma (n = 85) 24% | The family *Christensenellaceae* was associated with asthma and asthma associated intestinal metabolites (P = 0.03) | Poor |
| Chiu et al (2019)[45]; cross-sectional study  16S rRNA sequencing, V3-V4 region, Illumina HiSeq 2500 platform | Children between ages of 4-7 years with asthma, and healthy controls (n = 58) | Asthma; at 4‐7 years | 0 months (assessed in chidren with existing asthma + controls) | Physician‐diagnosed asthma;   Asthma diagnosis based on having has asthma, coupled with occurrence of recurrent wheeze in the last 12 months, or current use of asthma medication | Children with asthma (n = 34) 59% Healthy control (n = 24) 41% | **Children with asthma compared to healthy controls: Phylum**: lower *Firmicutes* (P = 0.009) **Species**: lower *Faecalibacterium, Anaerostipes, Eubacterium,* and *Roseburia* (P < 0.01), increase in *Clostridium* **Genus**: higher *Escherichia*, *Alistipes*, *Bilophila* and *Enterococcus*  **In healthy controls:**  Higher *Faecalibacterium* and *Roseburia* | Fair |
| Bannier et al (2019)[46]; prospective case-control study  16S rRNA, V3-V4 region | Children between the ages of 2-4 years with wheezing, and control subjects (n = 252)  Stool sample was taken at time of inclusion (n = 230)  Asthma Detection and Monitoring (ADEM) | Asthma; at 6 years | Approximation:  Between  48– 24 months | Based on results of the ISAAC Questionnaire, children with recurring asthma-like symptoms (experimental group) and 50 children with no respiratory symptoms (control group) were selected to participate in a two-month study with ICS. Exhaled biomarkers of inflammation/oxidative stress, as well as pulmonary function tests, were examined repeatedly. During the follow-up period, development of respiratory symptoms, lung function indices, and inflammatory biomarkers was monitored. Definitive diagnosis of asthma was made at the age of six years, based on several lung function measurements and respiratory symptoms. | True asthmatics (n=70) 35% Transient wheezers (n = 114) 56% Healthy controls (n = 46) 23% | **Children with asthma compared to healthy controls: Genus:** higher *Escherichia* (P = 0.02) and *Gemmigr* (P = 0.03), lower *Collinsella* (P = 0.01) and *Dorea* (P = 0.02) **Alpha diversity:** Microbial richness (OR 0.99 [95%CI 0.98-1.01]; P = 0.46) nor microbial diversity were significantly different (OR 1.01 [0.98 - 1.04]; P = 0.53) **Beta diversity:** No significant difference between transient wheezers and true asthmatics (P = 0.07) nor between preschool wheezers and healthy controls (P = 0.22) | Good |
| Galazzo et al (2020)[33]; cohort study  16S rRNA, V3 region, Illumina MiSeq, and Greengenes database (2011) | Newborn babies (n = 440);  at 5 weeks (n = 306) 13 weeks (n = 287) 21 weeks (n = 268) 31 weeks (n = 307) | Asthma; at 6–11 years | Approximation:  Between  65 –131 months | Physician-diagnosed asthma, in addition to any symptoms in previous 12 months (wheezing, shortness of breath, nocturnal awakening due to symptoms) | Asthma (n = 292) 66% | Lower *Lachnobacterium*, *Lachnospira*, and *Dialister* (P <0.001)  **Alpha diversity:** No significant difference between 5-13 weeks, with a gradual increase between 13-31 weeks and largest diversity seen at >31 weeks | Good |
| Patrick et al (2020)[34]; prospective cohort study  16S rRNA sequencing, V4 region, Hiseq Ilumina, Greengenes  database (2013) | Children from CHILD Cohort (n = 917); at 3 months and/or 1 year  Canadian Healthy Infant Longitudinal Development (CHILD) cohort | Asthma; at 5 years  *Outcome measured in those with stool sample processed at one year.* | 60 months | Clinical assessments by study physicians at age five years;  Diagnosis of asthma based on questionnaire data, clinical history, medical examination, and parent responses to ISAAC questionnaires. | Asthma (n = 63) 11% | **Children with asthma compared with children without asthma: At one year:** Lower *Faecalibacterium prausnitzii* (log2FC –1·57 to 1·77), *Ruminococcus bromii* (log2FC –2·07), and *Rikenellaceae* (family) (log2FC –2·59) and higher *Dialister* (genus) (log2FC 2·04; false discovery rate p<0·05)   **Alpha diversity:** At one year: decreased α-diversity relative abundance of bacterial taxa (only summarising those with at least 1·5 log2FC):  **Protective effects:** Having increased α-diversity at age one year protected from asthma (OR 0·68 [0·46–0·99]; p=0·046); | Good |
| Depner et al (2020)[35]; cohort study  16S rRNA sequencing, V4 region, Illumina MiSeq, and Greengenes database (2013); Fungal internal transcribed spacer region 1, UNITE dynamic database (2010) | PASTURE birth cohort (n =720); 2 months and 1 year | Asthma; at 6 years; | 70 months/  60 months | Parent reported, physician diagnosed Asthma based on at least once or recurrent diagnoses of obstructive bronchitis or asthmatic bronchitis. Atopic and non-atopic asthma based on presence or absence of sensitization to inhalant allergens (such as seasonal or perennial allergies) with specific IgE concentrations > 0.7 IU/mL-1 at six years. Lung function assessed through spirometry at six years.  **Asthma**: diagnosis by age six years | Asthma: (n = 53) 9% | **Children with asthma as compared to control subjects at age two months:** Lower *Bacteroides* and *Parabacteroides;* Higher *Enterococcus*  **Protective effects:** High relative abundance of *Bacteroides* and *Parabacteroides* and low relative abundance of *Enterococcus* at age two months was shown to have protective effects from both atopic and non-atopic asthma (OR 0·68 [0·49–0·95]; p=0·024);  **At age one year:** Lower *Roseburia*, *Ruminococcus*, and *Faecalibacterium* **Protective effects:** High relative abundance of *Roseburia*, *Ruminococcus*, and *Faecalibacterium* at age 1 yr and low relative abundance of *Enterococcus* at age 2 months was shown to have protective effects from non-atopic asthma (OR 0·62 [0·39–1·00]; p=0·048)  **Alpha diversity:** Positive correlation [r=0·70] for number of different bacteria genera; children with asthma had a difference in EMA at age 2 months and lower EMA at age 1 year than did children without asthma; | Good |
| Boutin et al (2020)[36]; cohort study  16S rRNA sequencing, V4 region, Illumina MiSeq, Greengenes database (2013) | Children from CHILD Cohort (n=837) (term >35 weeks); at 3 months | Recurrent wheeze and atopic wheeze; at 1 year | 9 months | Child Health Questionnaires and clinical evaluations conducted by study physicians at ages one, three, and five years.   **Recurrent wheeze:** experiencing more than two episodes of wheezing during first year of life | Recurrent wheeze and atopic wheeze at one year (n = 659) 79%  Recurrent wheeze (n= 142) 21%  Atopic wheeze (n = 45) 6% Both recurrent wheeze and atopic wheeze: (n = 16) 2% | C**hildren with recurrent wheeze, vs healthy children** *at three months:* Lower *Faecalibacterium*, *Lachnospira*, *Coprococcus*, and *Oscillospira* **Children with atopic wheeze, vs healthy children** *at 3 months:* Lower *Faecalibacterium, Lachnospira, Coprococcus, Roseburia, Blautia, Parabacteroides,* and *Ruminococcus*  ***Alpha diversity:*** *Increased α-diversity at age three months was protective of recurrent wheeze (OR 0·75 [0·6–0·95]; p=0·007) and atopic wheeze (OR 0·55 [0·1–0·90]; p=0·016);* | Good |
| Boutin et al (2021)[37]; cohort study  16S rRNA sequencing, V4 region, Greengenes reference database (2013)  ITS-2 rRNA gene sequencing, quantitative PCR (qPCR)-based total fungal load | Children from CHILD Cohort (n = 343); at 3 months at 1 year | Asthma; at 5 years | 57 months/  48 months | Parent reported, pediatrician diagnosed asthma, at age five years. Decision was based on a structured interview with parent or guardian.   **Asthma:** diagnosis made on basis of parent report of use of bronchodilator prescribed for episodes of coughing or wheezing, and use of prescribed daily controller medication, or frequent wheezing (more than three episodes within past year).  **Non cases/Possible Asthma:** less than three episodes of wheeze or coughing without colds, and no medication use. | **Asthma** (n = 33) 10.7%  ***Non-cases*** No asthma (n = 239) 77.6% Possible asthma (n = 33) 10.7% No phenotype available (n = 3) 1% | **In children with asthma at age five years, as compared to control subjects:** *Both three-month and 1 year sample S. cerevisiae* (ASV1) found to be an indicator from infants who developed inhalant atopy at age five years (stat = 0.882; P = 0.005), atopy at age five years (stat = 0.879; P = 0.005), or asthma at age five years (stat = 0.892; P = 0.005).  **In children who developed inhalant atopy at age 5 years as compared to control subjects (p < 0.05):** *3 months:* Lower *Malassezia;* Higher *Rhodotorula,* non*-albicans Candida 1 year of age:* Lower *Debaryomyces, Saccharomyces,* and *Candida;* Higher allergy-associated fungi such as *Alternaria*   Total fungal load was not significantly associated with health outcomes.  **Alpha diversity of mycobiota** 3-month stool sample: Increased alpha diversity associated with inhalant atopy. (p=0.21) 1-year sample: Decreased alpha diversity in 1-year samples associated with inhalant atopy.  **Beta diversity of mycobiota** 3-month stool sample: Significant differences associated with sensitization to inhalant allergens at age 5 years. 1-year: No difference | Good |
| Hsieh et al (2021)[41]; case-control study  16S rRNA sequencing V4, SILVA database[48] | Children 9 – 17 years  (n = 80) | Asthma and asthma severity; Children between ages 9-17 years. Mean age 14.0 (SD 2.0), with children with asthma slightly older than control individuals (p = 0.003) | 0 months (assessed in chidren with existing asthma + controls) | Home visits conducted to collect data on spirometry, anthropometry, and questionnaires administered to collect information about respiratory symptoms and asthma control. Asthma severity classified according to National Asthma Education and Prevention Program Expert Panel Report 3 guidelines[49]. Asthma control assessed over previous 4 weeks from home visit, using validated Spanish language versions of Childhood Asthma Control Test (ACT) for children 9-11 years, and the ACT for adolescents aged 12-17 years.  **Asthma:** physician diagnosis of asthma with asthmatic symptoms or asthma medication use within past year. **Asthma control:**  moderate to severe persistent asthma; Well-controlled asthma: score > 20 Persistent Asthma; Uncontrolled asthma: score < 20 | **Asthma (n = 40)** Persistent Asthma (n = 32) 80% Moderate to severe persistent asthma (n = 16) 40%  **Control (n = 40)** | **Children with asthma compared to control subjects** No difference reported  **Alpha diversity:** no difference **Beta diversity:** no difference (PERMANOVA comparison, p = 0.25) | Poor |
| Lee-Sarwar et al, (2022)[39]; prospective cohort study  16S rRNA sequencing V4, Illumina MiSeq, SILVA database[48] | Children from VDAART Cohort; Children with asthma  at 3 years (n = 110) | Asthma severity;  at 3 years | 0 months | Parental report of physician diagnosis of asthma or occurrence of recurrent wheeze in the child's first three years of life as determined by questionnaires administered postnatally, and every three months until child reached age three years. Asthma severity measured through proportion of questionnaires on which wheeze was reported.  **High wheeze:** reported wheeze on > 33% of questionnaires **Low wheeze:** reported wheeze on < 33% of questionnaires | High wheeze (n = 56) 51% Low wheeze (n = 54) 49% | (Spearman p = 0.43, P = 0.0002) **Positively associated with wheeze proportion** **Genera:** *Veillonella, Roseburia, Parasuterella, Ruminococcus (Ruminococcus gouvreauii group), Eubacterium (Eubacterium siraeum group), Clostridium (Clostridium* sensu stricto 1) **Class:** *Clostridia* UCG-014 **Family:** *Anaerovoracaceae* Family XIII AD30111 group  **Negatively correlated with wheeze proportion**  **Genera:** *Holdemanella, Senegalimossilia, Colidextribacter, Eubacterium fissicatena group* **Family:** *Lachnospiraceae (Lachnospiraceae* CAG-56), *Oscillospiraceae (Oscillospiraceae* UCG-003, *Oscillospiraceae* UCG-005), *Ruminococceae (Ruminococceae* CAG-352) **Alpha diversity:** Not associated with wheeze frequency (Shannon index: Pearson p = -0.12, P = 0.21; Simpson index: Pearson p = -0.10, P = 0.27) **Beta diversity:** Not associated with wheeze proportion (PERMANOVA P = 0.79) | **Poor** |
| Lee-Sarwar et al (2023)[38]; prospective cohort study  16S rRNA V4, Illumina MiSeq, SILVA database[48] | Newborn babies from VDAART Cohort; at 3-6 months (n = 265) at 1 year (n = 436) at 3 years (n = 506) | Asthma and asthma severity;  Between ages 0–6 yrs | Approximation:  Between  36–69 months | Parental report of physician diagnosis of asthma or occurrence of recurrent wheeze as determined by questionnaires administered postnatally, and every three months until child reached six years of age. Asthma severity measured through proportion of questionnaires on which wheeze was reported. Impulse oscillometry performed at yearly visits, at ages four years, five years, and six years, to validate asthma diagnosis. **Early asthma:** Physician diagnosed asthma, or reported recurrent wheeze by age three years **Active asthma:** Physician diagnosed asthma, report of wheeze and/or asthma medication used between five and six years **Transient asthma:** Early asthma, without active asthma at six years | Early asthma (n = 185) 28%  Mutually Exclusive: Active asthma (n = 115) 19% Transient asthma (n = 90) 15% No asthma (n = 405) 66% | **Children with transient wheeze and early asthma as compared to those without** *3 – 6 months:* Lower *Staphylococcus* (FDR < 0.05); Lower *Bacteriodes* (FDR < 0.05) *1 or 3 years:* No fecal genera associations found with any asthma phenotype (FDR > 0.05)  **Children in a low-asthma-risk cluster found to have:** *3–6 months and one year:* Higher *Bacteriodes* (p = 0.04)  **Alpha diversity:** not associated with any asthma phenotype (p > 0.05)  **Beta diversity:** At age three years associated with active asthma (PERMANOVA F = 2.50, p = 0.004) | Good |
