# Supplementary material for: Link between gut microbiota dysbiosis and childhood asthma: Insights from a systematic review
Source: J Allergy Clin Immunol Glob. 2024 Jun 12;3(3):100289. doi: 10.1016/j.jacig.2024.100289 (PMC11298874; doi:10.1016/j.jacig.2024.100289)
Supplement: Supplementary Tables [file mmc2.docx]

# APPENDIX 1

The following tables include a record of the study quality assessments made according to the Newcastle-Ottawa Scale for the human cohort studies (Table 1), human cross-sectional studies (table 2), and human case-control studies (table 3).

| **Table 1. Newcastle–Ottawa Scale for the human cohort studies.** | | | | | | | | | | |
| --- | --- | --- | --- | --- | --- | --- | --- | --- | --- | --- |
| **Study** | **Representative-ness of exposed cohort** | **Selection of non-exposed cohort** | **Exposure ascertain-ment** | **Demonstration that outcome of Interest was absent at study onset** | **Comparability of cohorts based on study design or analysis** | **Outcome assessment** | **Was follow-up time long enough for outcome to occur?** | **Cohort follow-up adequacy** | **Total** | **Study quality** |
| Fujimara et al. (2016)[40] | 1 | 1 | 0 | 1 | 1 | 0 | 1 | 1 | 6 | Fair |
| Stokholm et al. (2018)[31] | 1 | 1 | 1 | 1 | 1 | 1 | 1 | 1 | 7 | Good |
| Lee-Sarwar et al. (2019)[32] | 1 | 0 | 1 | 0 | 1 | 1 | 0 | 1 | 5 | Poor |
| Galazzo et al. (2020)[33] | 1 | 1 | 1 | 1 | 1 | 1 | 1 | 1 | 7 | Good |
| Patrick et al. (2020)[34] | 1 | 1 | 1 | 1 | 1 | 1 | 1 | 1 | 7 | Good |
| Depner et al. (2020)[35] | 1 | 1 | 1 | 1 | 1 | 1 | 1 | 1 | 7 | Good |
| Boutin et al. (2020)[36] | 1 | 1 | 1 | 1 | 1 | 1 | 1 | 1 | 7 | Good |
| Boutin et al. 2021[37] | 1 | 1 | 1 | 1 | 1 | 1 | 1 | 1 | 7 | Good |
| Lee-Sarwar et al. (2022)[39] | 1 | 0 | 1 | 0 | 1 | 1 | 0 | 1 | 5 | Poor |
| Lee-Sarwar et al. (2023)[38] | 1 | 1 | 1 | 1 | 1 | 1 | 1 | 1 | 7 | Good |

| **Table 2. Newcastle–Ottawa Scale for human cross-sectional studies.** | | | | | | | | | |
| --- | --- | --- | --- | --- | --- | --- | --- | --- | --- |
| **Study** | **Representativeness of sample** | **Sample size** | **Non-Respondents** | **Exposure ascertainment** | **Comparability of subjects in different outcome groups based on study design or analysis. Confounding factors were controlled** | **Outcome assessment** | **Statistical test** | **Total** | **Study quality** |
| Chiu et al. (2019)[45] | 1 | 0 | 1 | 2 | 1 | 1 | 1 | 7 | Fair |

| **Table 3. Newcastle–Ottawa Scale for the human case-control studies.** | | | | | | | | | | |
| --- | --- | --- | --- | --- | --- | --- | --- | --- | --- | --- |
| **Study** | **Outcome Definition Adequacy** | **Representative-ness of Cases** | **Control selection** | **Control definition** | **Comparability of Cases and Controls based on Design or Analysis Ascertainment** | **Exposure Ascertainment** | **Method of ascertainment for cases and controls was the same** | **Non-response rate** | **Total** | **Study quality** |
| Arrieta at al. (2015)[44] | 1 | 1 | 0 | 0 | 2 | 1 | 1 | 0 | 6 | Poor |
| Stiemsma et al. (2016)[42] | 1 | 1 | 1 | 1 | 2 | 1 | 1 | 0 | 8 | Fair |
| Arrieta et al. (2018)[43] | 1 | 1 | 1 | 1 | 2 | 1 | 1 | 1 | 9 | Good |
| Bannier et al. (2019)[46] | 1 | 1 | 1 | 1 | 2 | 1 | 1 | 1 | 9 | Good |
| Hsieh et al. (2021)[41] | 1 | 1 | 0 | 1 | 1 | 1 | 1 | 0 | 6 | Poor |
